# Data for whom? Experiences and perceptions of a perinatal eRegistry in two hospitals in Mtwara region, Tanzania

**DOI:** 10.1136/bmjgh-2024-016765

**Published:** 2024-11-20

**Authors:** Jil Molenaar, Amani Kikula, Yusufu Kionga, Hassan Tearish Berenge, Lenka Benova, Josefien van Olmen, Claudia Hanson, Muzdalifat Abeid, Andrea Barnabas Pembe

**Affiliations:** 1 University of Antwerp, Antwerpen, Belgium; 2 Institute of Tropical Medicine, Antwerpen, Belgium; 3 Muhimbili University of Health and Allied Sciences, Dar es Salaam, Tanzania, United Republic of; 4 Ifakara Health Institute, Dar es Salaam, Tanzania, United Republic of; 5 Karolinska Institute, Stockholm, Sweden; 6 London School of Hygiene and Tropical Medicine, London, UK; 7 The Aga Khan University - Tanzania, Dar es Salaam, Tanzania, United Republic of

**Keywords:** Maternal health, Accountability, Public Health, Qualitative study, Internet

## Abstract

**ABSTRACT:**

**Introduction:**

Digital data systems have the potential to improve data quality and provide individual-level information to understand gaps in the quality of care. This study explored experiences and perceptions of a perinatal eRegistry in two hospitals in Mtwara region, Tanzania. Drawing from realist evaluation and systems thinking, we go beyond a descriptive account of stakeholders’ experiences and provide insight into key structural drivers and underlying social paradigms.

**Methods:**

We carried out 6 weeks of focused ethnographic observations at the labour wards of the two hospitals and 29 semi-structured qualitative interviews with labour ward staff, as well as with administrative and managerial stakeholders at hospital, district and regional levels. Multi-stage reflexive thematic data analysis was carried out.

**Results:**

We provide an in-depth account of the day-to-day functioning of the eRegistry in the two hospitals, including both aspects of positive change and key challenges with its integration into routine documentation duties. Experiences with and perceptions of the eRegistry were inextricably linked to broader systemic constraints relating to staffing, workload and infrastructure. A key underlying theme shaping the way people engaged with the eRegistry was the notion of data ownership: the presence or absence of a feeling of being responsible, involved and in control of data.

**Conclusion:**

Some of the key systemic challenges in recording accurate, timely information about women and their babies are not solved by digital tools. Our findings also underline that when healthcare workers feel that data are not primarily for them, they document only for reporting purposes. The eRegistry increased a sense of data ownership among the nurse-midwives directly involved with data entry, but the potential for promoting and supporting data use feedback loops for improvement in care provision remained largely untapped. Our findings highlight the importance of local relevance and ownership in digitisation of routine health information systems.

**WHAT IS ALREADY KNOWN ON THIS TOPIC**

- Routine health information systems (RHIS) in low- and middle-income countries are key for country-led health system strengthening.
- Digital data systems are widely viewed as having potential to improve data quality and provide more fine-grained, individual-level information to better understand gaps in the quality of care.

**WHAT THIS STUDY ADDS**

- Through changes in the modalities of data collection and feedback loops, the eRegistry shifted the sense of data ownership during the study period.
- Maternal and neonatal health data are entangled with human interactions and context-specific drivers. Therefore, boosting perceived relevance and ownership of data is just one piece of the complex puzzle of promoting productive data use cultures.

**HOW THIS STUDY MIGHT AFFECT RESEARCH, PRACTICE OR POLICY**

- Our findings highlight that we must avoid ‘techno-utopian’ views of digital technologies as straightforward ways to fix problems in RHIS. Systemic embeddedness is required to produce accessible, relevant data that can be used for iterative quality improvement in health services.

## Background

 Despite considerable progress, the perinatal period continues to be an unacceptably dangerous period for women and babies in many parts of the world. The burden of maternal and neonatal mortality is particularly high in sub-Saharan Africa, with more than half of the world’s maternal deaths occurring in this region.^1 2^ Health data are considered critical to understand the magnitude of this burden and track progress. Many international maternal and neonatal health monitoring initiatives are still heavily reliant on population-based household surveys like the Demographic and Health Surveys (DHS). However, indicator estimates can also be derived from routine health information systems (RHIS), which aggregate data directly from health facilities.^3 4^ RHIS, also referred to as health management information systems (HMIS), can facilitate the use of health data across all health system levels to inform service planning and provision.^5^

There have been repeated calls to strengthen RHIS in low- and middle-income countries (LMICs) over the past decades, as these systems are considered foundational to promoting and guiding long-term, country-led health system strengthening.^6 7^ Yet, RHIS building remains challenging in many LMICs.^8^ Data from RHIS are frequently described to be of poor quality, owing to problems with completeness, timeliness and accuracy of reporting.^3 9^ Even when data are complete, timely and accurate, this does not guarantee they will be used. Indeed, other root causes of poor RHIS functioning relate to accessibility of data, as well as limited human resource availability and capacity to analyse and interpret data, contributing to the absence of a ‘data use culture’.^10 11^

Many LMICs, including Tanzania, are currently pursuing digitisation of their RHIS using platforms like District Health Information Software 2 (DHIS2).^12 13^ DHIS2 is used in over 70 countries globally, supporting routine health data management and storage, as well as data analysis, presentation and use.^14^ In most countries, paper-based registers and tools continue to be used to collect data at the facility level, which are subsequently aggregated and entered into the DHIS2 system at the district level. Perhaps partly as a result, there is often a heavy focus on reporting data upwards in DHIS, rather than on the generation of data for facility-level use.^10^ In addition to DHIS2, many LMICs have seen the introduction of digital systems at individual health facilities that collect data for both clinical and managerial purposes. Some electronic health record systems primarily aim to improve service delivery through systemised collection of patient health data, while others are more focused on financial accountability or human resource management.^15 16^

When the Tanzanian RHIS was first established in the early 1990s, it was paper-based at all health system levels.^17^ Between 2011 and 2014, DHIS2 was gradually introduced in Tanzania to enter monthly summary information digitally at the district level.^17^ The Tanzanian government implemented a Data Dissemination and Use Strategy for its health sector in 2015, as well as a National Data Quality Guideline in 2016.^17 18^ In its 2021 Health Sector Strategic Plan V, the Ministry of Health (MoH) expressed the ambition that ‘the HMIS should become the basis for evidence-based planning and management from the lowest level to the top level in the health sector’.^18^ However, data quality issues remain in Tanzania’s routine health data. An analysis of emergency obstetric and neonatal care indicators from Tanzanian DHIS2 data (2016–2020) showed that although data completeness was high at 98%, rates of obstetric complications and several obstetric interventions were implausibly low, indicating under-reporting.^4^ Moreover, facility- and district-level data use remains limited. A cross-sectional study in 11 Tanzanian districts found that there was a widespread sentiment among facility healthcare workers (HCWs) and district officials that data are collected merely for reporting purposes, and that utilisation of RHIS data was limited at facility and district level.^19^

This study was linked to the Action Leveraging Evidence to Reduce perinatal morTality and morbidity (ALERT) project. ALERT was a hospital maternity-based quality improvement and implementation science project implemented across 16 hospitals in Benin, Malawi, Tanzania and Uganda.^20^ To support its codesigned competency-based training and quality improvement approach, the ALERT project implemented a perinatal eRegistry. In addition to providing indicators of process and outcomes for the ALERT evaluation, the eRegistry was also envisioned to enable regular feedback to maternity care providers and enhance facility-level data use.^20^ Women’s data were collected in electronic patient records (EPRs) on tablets, thus providing individual data in real-time. To facilitate easier data interpretation by hospital teams, run charts (plots displaying data in a time sequence) were shared by the ALERT team on a monthly basis. Linking to the quality improvement cycles taking place at the hospitals, the run-charts focused on target areas for improvement such as the completeness of obtaining admission data and the consistency of fetal monitoring. During quarterly field visits, data were shared with data collectors, hospital managers, as well as district and regional officers. The ALERT project employed realist evaluation as a theory-driven evaluation approach. Drawing from work by Pawson and Tilley,^21^ realist evaluation aims to decipher the ‘black box’ between an intervention and its outcomes, focusing on understanding the connections between the intervention, actors, outcomes, contexts and mechanisms of change.^22^

**Figure 1:**
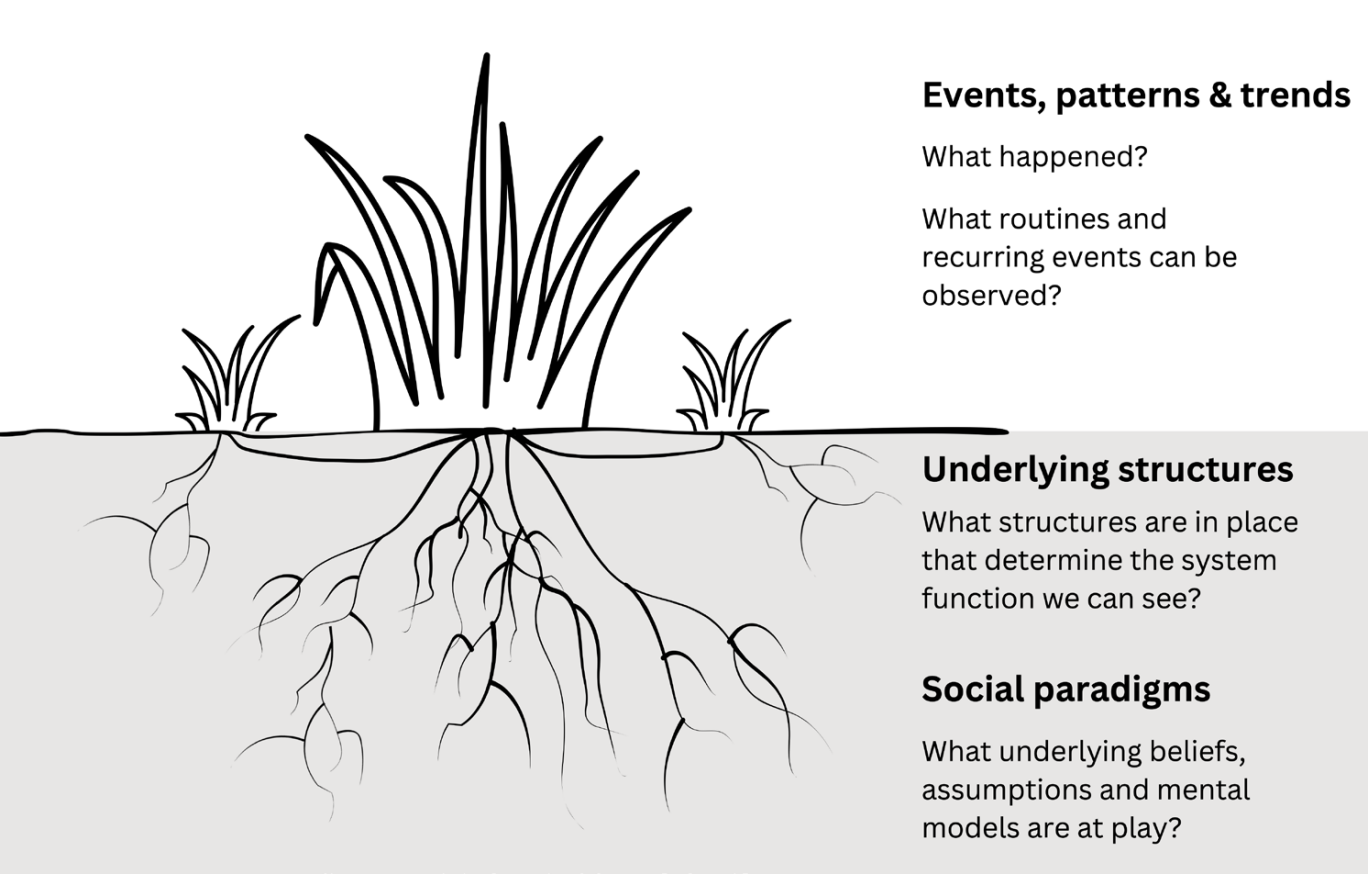
A grass plant as a system representation. From above, one might only see the larger outshoots (events). Closer to the ground, side shoots are visible (patterns and trends). The plant is anchored by its roots, some closer to the surface (underlying structures), some extending deeper into the soil (social paradigms).

Our study objective was to explore experiences and perceptions of the perinatal eRegistry in two hospitals in Mtwara region, Tanzania. Through in-depth interviews and observations, we aimed to go beyond a descriptive account of stakeholders’ experiences and perceptions, and provide insight into key structural drivers and underlying social paradigms. The language and inquiry rationale of the ALERT realist programme theory were relevant to this aim, as realist evaluation underscores how we must interrogate social systems and structures in order to understand how the outcomes of interventions or programmes have come about.^21^ By recognising how outcomes comprise both intended and unintended consequences, realist evaluation highlights complexity, resonating with the guiding principles of systems thinking.^23^ We draw on key concepts from systems thinking, too, specifically the commonly made distinction between visible manifestations of a system—events—versus the patterns, structures and social paradigms that lie under the surface and are prone to go unseen.^24^ This is often visualised using an iceberg model, with events presented as the tip of the iceberg.^24^,^26^ We opted to present the system as a grass plant, instead, as we consider a plant more universally recognisable than an iceberg, as well as a better representation of the dynamic nature of the system.^27 28^ Our conceptual framework is illustrated in figure 1.

## Methods

### Study setting

Data collection took place in Mtwara region in Southern Tanzania, over 500 km south of Tanzania’s largest city Dar es Salaam. Mtwara is a rural region with a population of around 1.6 million in 2022.^29^ The region is predominantly agricultural, and most of the population engages in subsistence farming. Hospitals A and B are among the three hospitals in Mtwara region where the ALERT project implemented the perinatal eRegistry. Key characteristics of hospitals A and B are summarised in table 1.

**Table 1:** Hospital characteristics

Hospital A	Hospital B
Faith-based, not-for-profit facility functioning as a regional referral hospital.	Typical public rural district hospital.
Around 2000 annual deliveries.	Around 3000 annual deliveries.
Comprehensive emergency obstetric and newborn care (CEmONC) services are offered. There is a neonatal intensive care unit with incubators, baby heaters and baby—continuous positive airway pressure machines.	CEmONC services are offered. There is a neonatal care unit (not on intensive care unit level).
Labour ward staff include a gynaecologist, a medical doctor, intern doctors and nurse-midwives at certificate or diploma level. During a typical work shift there are 2–3 nurse-midwives at the labour ward.	Labour ward staff include a medical doctor and nurse-midwives at certificate or diploma level. During a typical work shift there are 1–2 nurse-midwives who are responsible for both the antenatal and labour ward.
Several delivery rooms offering more privacy. Running water and electricity are almost always available.	A single delivery room. Running water and electricity are often not available. There is a generator for the operating theatre.

In both hospitals, there is a multitude of systems to collect and report data. HMIS data requested by the MoH are collected on paper-based registers known by the Swahili abbreviation MTUHA, and aggregated on tally sheets before entry into DHIS2 software. The antenatal care (ANC) card is a printed card that a woman is supposed to bring every time she visits a healthcare facility, including for childbirth. On admission, various locally printed forms and observation charts, as well as the partograph for labour monitoring, form part of a woman’s clinical patient file. In hospital A, a locally developed electronic health information system for clinical and hospital management purposes is used in addition to paper-based documentation. Finally, a wide range of more informal documentation is present in the labour wards of both hospitals. This includes staff attendance registers; surgery record books; doctor’s rounds notebooks; shift hand-over notes; stillbirth registers; referral books; and drug-specific registers.

The perinatal eRegistry implemented by the ALERT project functioned as an additional, parallel data system. The main motivation behind introducing a parallel system was that it allowed for collection of individual-level data for the project’s effect evaluation. In addition, data were used to inform quality improvement. The typical aggregated totals reported in the HMIS were not suitable for such purposes. The eRegistry was in use from June 2021 until February 2024. Data for the eRegistry had to be extracted from several sources, including the ANC card, HMIS register book (MTUHA), partograph and other clinical notes. Initially, all nurse-midwives working at the labour ward participated in eRegistry data entry and received a small monetary compensation for this. However, in an effort to improve data quality, this strategy evolved after 6 months to designate two labour ward staff for data collection per facility. These nurse-midwives continued to do their normal clinical duties and were compensated by ALERT for the extra time spent on the eRegistry duties.

### Study design, sampling and data collection

This study adopted a qualitative approach, incorporating both focused ethnographic observations and semi-structured qualitative interviews. Data collection took place in October to December 2023. The first author spent 3 weeks at the labour ward of each hospital, developing a rapport with the staff to facilitate spontaneous discussions and observations. A total of approximately 230 hours of observations were conducted, including not just weekday shifts but also several shifts during weekends, evenings and nights. Observations included a focus on understanding day-to-day routines in documentation and reporting duties; division of responsibilities; overlap between various documentation types; and the ways maternity care providers communicated about documentation tasks. Detailed field notes were compiled from these observations, digitalised regularly for analysis.

In addition, 29 semi-structured interviews were carried out. Using purposive sampling, we selected participants to capture diverse experiences with and perceptions of the perinatal eRegistry. Interview participants included: (1) nurse-midwives contracted by ALERT to manage eRegistry data entry; (2) other labour ward HCWs; and (3) administrative and managerial stakeholders at hospital, district and regional levels. Out of these interviews, 10 were conducted in hospital A, 11 in hospital B, 5 were conducted at the district level and 3 at the regional level. For more details on participant characteristics, see table 2.

**Table 2:** Interview participant characteristics

Current professional role	Gender		Age group				
	Man	Woman	25–29	30–34	35–39	40–44	45+
Labour ward HCWs (n=18)	9	9	11	5	0	1	1
Nurse-midwives n=14							
Maternity-in-charges (nurse-midwives) n=2							
Medical doctor or doctor in training n=2							
Managers (n=11)	5	6	0	3	1	3	4
Hospital-level managers n=3							
District-level managers n=5							
Regional-level managers n=3							

Semi-structured interviews took place in locations offering audio-visual privacy, including offices, meeting rooms and unused consultation rooms. The interviews were conducted following topic guides, with tailored topic guides for the three different groups of interviewees (see online supplemental file 1). Topics included in the guide were general perceptions of the eRegistry; changes in experiences over time; responsibilities and feedback mechanisms; data completeness and quality; and perceived long-term relevance and feasibility of the perinatal eRegistry. In addition to eRegistry specific questions, the topic guides also contained questions on RHIS data more generally. The topic guides were informed by findings from previous qualitative research on collecting and reporting maternal and neonatal health data in Southern Tanzania,^30^ as well as in other low- and lower-middle income country settings.^31^ Field notes were reviewed on a daily basis, and preliminary findings guided further data collection, including additions and adjustments to the interview topic guides. Two research assistants fluent in Swahili (HTB and YK) led the interviews at hospitals A and B, respectively, to ensure linguistic accuracy and cultural sensitivity; JM was present at all interviews and took notes. The interviews were audio-recorded (average recorded length 58 min) and transcribed verbatim.

### Data analysis

Over the course of data collection, regular review of field notes and interview transcripts informed initial analyses. When data collection was concluded, debrief discussions among members of the research team facilitated collaborative interpretation. After completion of transcription of all interviews, initial codes were identified by JM through refamiliarisation with the whole data set. Subsequent reflexive thematic analysis was done in NVivo V.1.7.1 software.^32^ Following double-coding of a subset of interview transcripts by JM and AK, a shared coding tree was developed. This codebook was used for focused coding of all transcripts and field notes in NVivo by JM. Through discussions within the author team, theoretical coding evolved to harness the system categorisations illustrated in figure 1. The distinction between visible and invisible manifestations of the system was considered to add explanatory power to the findings and was used to structure the write-up of the analysis. This paper presents a subset of the findings from this qualitative study—other key themes, notably relating to the power dynamics shaping the collection of maternal and neonatal health data, will be reported elsewhere. No translations were done prior to coding (transcripts were in Swahili, field notes were in English), but a selection of relevant quotes and extracts per code was translated into English to facilitate discussion within the author team and guide write-up. Co-authors who are native Swahili speakers cross-checked the translations.

## Findings

We present our findings thematically in three sections, following our conceptual framework (figure 1). We thus begin with describing visible characteristics of the system, before moving ‘deeper’ to understand underlying structures and mental models. Concretely, we start with a description of key events, patterns and trends: the general functioning of the eRegistry in the two hospitals. This includes a summary of stakeholders’ perceptions of positive change, as well as of some key challenges. Second, we discuss underlying structures, namely the broader systemic constraints which shaped experiences with and perceptions of the eRegistry. Third, we focus on the key underlying social paradigm which emerged from the findings: a sense of data ownership.

### Events, patterns and trends: general functioning of the eRegistry

The combination of observations and interviewees’ accounts provided a comprehensive insight into the day-to-day functioning of the eRegistry in the two hospitals. When asked about how the perinatal eRegistry changed the way information about mothers and babies was reported, interview participants discussed various aspects of positive change. Being a digital solution, the eRegistry was perceived by many as progress by definition. Particularly the labour ward HCWs, who were often relatively young, saw the introduction of tablets as a sign of modernity and welcomed it with genuine enthusiasm. Paper-based documentation ‘easily gets lost’ (IV14, nurse-midwife) and ‘can just be blown away by the wind’ (IV6, nurse-midwife), whereas digitised information was considered more permanent and reliable. Digitisation can also improve accessibility of data:

Accessibility of manually recorded information is difficult […] For example, right now, if you ask me about a certain month and how many patients had pre-eclampsia, it means you’ve given me an assignment. I will have to look for the books (registers) and search for the information. It’s different from electronic searching. (IV26, maternity-in-charge)

Particularly among district and regional-level stakeholders, improved access and easier sharing of information were also considered to have positive implications for data use across geographical locations, allowing for comparative analyses: ‘now we could even compare information from different places’ (IV15, district-level manager).

Besides some inherent practical advantages of digitised data systems, there were also specific characteristics of the eRegistry which were perceived positively. Maternity staff who were actively involved with data entry in the eRegistry underlined that because the system flagged missing information, ‘there is no way to skip’ (IV11, intern doctor). As a result, there was an increased focus on timely completeness of the paper-based documentation in order to minimise missingness in the data entered in the eRegistry. The eRegistry was considered an intelligent system because of its capacity to detect inconsistencies and unlikely numbers: ‘it automatically corrects some errors’ (IV12, nurse-midwife).

In addition, the eRegistry was described as collecting more fine-grained information. In contrast with the monthly aggregation of paper-based documentation for DHIS2 entry, the eRegistry was continuously up-to-date. Access to real-time information was thus perceived as a positive change:

This information, you can even get it daily. That is, how many people received care, you can even look at those with complications and how they were treated and their progress. You can easily find them rather than going back to the register. (IV15, district-level manager)

The eRegistry data was also more fine-grained because it collected individual-level data, rather than aggregated totals. It was considered valuable that individual data for each woman followed her entire clinical journey: ‘It talks about the mother from when she enters until she exits and shows if there are any challenges’ (IV24, hospital-level manager). The format in which data were requested in the eRegistry made intuitive sense to nurse-midwives, ‘because they are arranged in a complete series of care’ spanning the antenatal, intrapartum and postpartum periods (IV16, nurse-midwife).

There were challenging aspects of the day-to-day functioning of the eRegistry as well. The most visible, surface-level issues related to technological challenges. The eRegistry tablets in both hospitals were physically damaged, and in hospital A, the team had gone without the tablet for an extended period of time as it had to be sent for repair. Interviewees noted how ‘the overall maintenance of the tablets’ posed a challenge for long-term sustainability (IV16, nurse-midwife). In addition, data synchronisation was sometimes hampered by poor internet connectivity. Particularly in hospital B, the internet connection could be rather unstable, and ‘the problem of the internet misbehaving’ led to frustration and complaints (IV21, nurse-midwife).

Delving a little deeper, another key trend was the limited extent to which the eRegistry was incorporated into routine documentation duties at the labour wards of both hospitals. It was originally envisioned that all labour ward staff would enter the data as part of their daily work routine. However, at the time of this qualitative study, just two nurse midwives per facility were in charge of entering eRegistry data. Observations at the two hospitals highlighted that this was typically done either prior to or at the end of their shifts. They would gather the various paper-based documents into small piles and enter them in one sitting. When the designated nurse-midwives had been away or had not found time for data entry these piles were higher, as they would have to catch up with the backlog. As such, eRegistry data entry was clearly an additional task: ‘it’s like documentation doubling, making the work a little harder’ (IV9, maternity-in-charge). Moreover, supervision of and support for the eRegistry data entry, as well as guidance in interpreting data trends, remained reliant on the ALERT team. Although run-charts of key indicators were shared with data collectors and managers at hospital, district and regional level on a monthly basis, the data feedback process was not fully embedded within existing systems such as Maternal and Perinatal Death Surveillance and Response meetings.

### Underlying structures: systemic constraints

Experiences with and perceptions of the eRegistry cannot be isolated from broader systemic constraints. The eRegistry was implemented in what many described as *mazingira magumu*—a difficult (work) environment—and therefore faced similar challenges as routine paper-based documentation, as well as a number of specific technical barriers. Central to many stakeholders’ accounts were the issues of staffing and workload. Timely, complete data collection can be near impossible in the context of staff shortage:

If you find that the ward or facility does not have enough staff, when you encounter a situation of serving a mother with childbirth complications, or if multiple mothers want to give birth at the same time, it can be a source of missing information again and again. (IV1, regional-level manager)

At hospital B, it was typical for a single nurse-midwife to be responsible for providing care at both the antenatal and labour wards. A nurse-midwife at this hospital explained that ‘if the healthcare provider is just one, the workload becomes heavy […] because as a maternity ward, we have a lot of data’ (IV18). Indeed, during busy shifts it was common for care providers not to have time for documentation duties at all, meaning it had to happen post-facto.

At both hospitals, a plethora of notebooks, register books, charts and sheets were used. Searching for missing paper-based documentation was a common feature of ward activities, particularly at hospital B. Care providers could frequently be observed to get frustrated when looking for a woman’s ANC card or admission notes in the chaotic flurry of papers spread across the ward. As eRegistry data entry depended on compilation of information from various sources, chasing down this information could be a time-consuming task:

But another issue is the unavailability of data. Sometimes data are not available, they are lost. They are not really lost, but they’re troublesome to find. You have to search for them a lot. (IV10, nurse-midwife)

Especially when eRegistry data entry was delayed, it was common that data would end up being incomplete ‘because the woman has already been discharged and has left with the card, now where else will you get her information?’ (IV20, nurse-midwife). In addition, missing information was frequent for women arriving without an ANC card—either because they had not attended ANC or because the card was lost—as well as for referral patients:

Referrals come and are left without a card, there is no partograph. So you arrive and go in for a caesarean, sometimes you don’t [even] know who brought the patient. So I am following up on that information or details […] those are the challenges we sometimes face. (IV5, nurse-midwife)

Infrastructural challenges also impacted the functioning of the eRegistry. As a digital solution, the eRegistry was more vulnerable to technical hiccups and dependent on power supply:

If there’s no electricity now, it won’t be there until evening. […] The tablet has no power, so you find that information is not entered on time. (IV21, nurse-midwife)

As such, structural challenges that impact care provision more generally also posed difficulties in the eRegistry implementation.

### Data ownership

An underlying theme shaping the way people perceived and engaged with the eRegistry was the notion of data ownership. We define ownership here not as legal possession, but rather as a feeling of being responsible, involved and in control. The presence or absence of a sense of data ownership was a key driving force of the way people thought about health data more generally, and the eRegistry specifically.

Our findings highlighted that the sense of ownership of paper-based data among nurse-midwives was typically limited. A nurse-midwife described how prior to her involvement with the eRegistry, her documentation duties were limited to a habitual inputting of data into the paper-based MTUHA register:

Honestly, I wasn’t following those record-keeping matters. Because, you see, I wasn’t in charge, I was just an ordinary worker, and I was mainly in the postnatal ward. So I was just doing my duties that concerned me, inputting into MTUHA what was required, I wasn’t following other things. (IV20, nurse-midwife)

Many interviewees echoed the sentiment that data-related duties were of minimal importance compared with clinical duties: ‘my responsibilities are to ensure that the mother and her child have crossed safely, but these record-keeping tasks belong to someone else’ (IV12, nurse-midwife). Absence of a sense of ownership went hand in hand with documenting for reporting purposes only:

Low-level workers, if they are not given a sense of ownership, they will create [documentation] not for their own learning, they will create just for reporting. They will strive for accuracy or lack of errors, not for themselves but for reporting purposes only, so that they will not be burdened with redoing it. (IV26, maternity-in-charge)

Nurse-midwives’ active role in data management for the eRegistry seemed to increase their sense of data ownership. As eRegistry data entry entailed triangulation of data from MTUHA, the partograph, admission forms and sometimes doctor’s notes, it required nurse-midwives to reconstruct a woman’s entire journey of care. Some of the nurse-midwives who were directly involved with the eRegistry indicated that this could be empowering and increase willingness to act on data trends:

At first, I thought it was just about entering numbers, and the job would be done, but it’s not like that. […] When you encounter a system that provides you with something, you should also consider from the other side, like I said before. So, you can sit down and prepare your report through the eRegistry, stating that for this particular issue, I need to conduct research or maybe submit a proposal based on one, two, three things I saw in these data. (IV5, nurse-midwife)

The ALERT team’s feedback on data trends directly to care providers was also described as allowing for quicker learning without higher-level intervention:

It helps us to make quick changes without relying on hospital meetings. So there are those quick measures to implement, even if the government hasn't convened meetings and spotted certain weaknesses. (IV21, nurse-midwife)

Therefore, it appears the eRegistry increased the sense of data ownership among the nurse-midwives who were directly involved with the project.

However, this sense of increased ownership remained limited to a relatively small group of individuals. While the decision to involve fewer nurse-midwives had a positive impact on data quality, some of the staff who had initially been involved resented the switch:

Now those on the side-lines feel like it’s someone else’s job. Initially, there was a morale that we all share in recording and understanding, which made it easier to use the system. However, when it was taken over by a few, even the morale to use the systems decreased. (IV12, nurse-midwife)

Most interviewees recommended that it was better to involve all care providers on the ward: ‘I would wish that more people would participate in this, because many people are also involved in caring for the patients’ (IV29, medical doctor).

Moreover, although most interviewees viewed the long-term potential of the eRegistry positively, it clearly remained a project initiative that was heavily reliant on external support and leadership. Neither care providers nor leaders at the hospital, district and regional level had direct access to the data, which led them to feel the data were not primarily for them. A maternity-in-charge suggested shifting the ownership to the hospital management:

Now it’s necessary for the hospital to bear the responsibility. I suggest that at the end of the day, this project should come from within … [otherwise] we can’t transition from using manual to electronic systems for the hospital. Now the research is complete, they’ve given us the results. […] So for now, let’s start returning ownership to the hospital administrators themselves. (IV26, maternity-in-charge)

Local-level ownership was thus perceived by many as a prerequisite for long-term use of digital systems.

## Discussion

This study explored different stakeholders’ experiences with and perceptions of a perinatal eRegistry in Mtwara region, Tanzania. Although the eRegistry was implemented in the context of a quality improvement and implementation science project, the qualitative insights have broader relevance for ongoing efforts to digitise Tanzania’s RHIS. With regards to the positive aspects of people’s experiences with the eRegistry, this study highlighted that digital solutions have notable potential to improve health data quality and use. The eRegistry was considered modern, secure and easy to work with, and it was perceived to facilitate accessibility and sharing of data. In addition, the individual-level data collected in systems like the eRegistry allow health workers to follow patients along the continuum of care and enable monitoring of sub-national coverage gaps in high-quality care provision.^33 34^

However, the findings also underline that the ways in which digital technologies like the eRegistry are adopted or resisted are far from simple. A significant proportion of medical and public health literature take what has been referred to as a ‘techno-utopian’ or ‘solutionist’ stance on digital health technologies, presenting these technologies as straightforward ways to fix problems.^35^ Yet, social science scholars have critiqued the digital health movement for focusing too much on the technology and too little on the complex ways in which technologies are entangled with human interactions and context-specific drivers.^36 37^ A systematic literature review of EPR research identified examples of widespread disruptions of professional routines, non-integrated EPR systems with continued dependence on paper, and abandoned EPR systems.^38^ In view of such experiences, there is a growing recognition that systems must have a sociotechnical design, based on the principle that the introduction of a technology is a social, context-specific process.^39 40^ In the case of tools like the eRegistry, a narrow focus on how digitisation might improve data quality risks glossing over the systemic drivers that shape people’s ability and motivation to collect and report routine maternal and neonatal health data.

We found that experiences with and perceptions of the eRegistry were inseparable from overarching systemic constraints related to staffing, workload and infrastructure. This resonates with WHO guideline recommendations on digital interventions for health system strengthening, which stressed how digital health ‘still shares many of the underlying challenges faced by health system interventions in general’ such as infrastructural limitations and human resource shortages.^41^ Some of the most pertinent challenges in recording accurate, timely information about women and their babies are not solved by digital tools. Across diverse low- and lower-middle income country contexts, availability of staff has been recognised as a key prerequisite and determinant of capacity and motivation to document and report data on maternal and neonatal health indicators.^31^ Specific issues affecting digital technologies such as poor internet connectivity, inadequate power supply, and malfunctioning or broken devices can add an extra layer of difficulty in conditions of scarcity and precarity.^42^,^44^

In our study, the presence or absence of a sense of data ownership emerged as a key driving force of the way people engaged with health data more generally, as well as the eRegistry specifically. We conceptualise the feeling of being responsible for and in control of data as a social paradigm—a ‘root cause’ that underlies people’s actions. Our findings underline that when HCWs feel that data are not for them, they document only for reporting purposes. Indeed, it has been noted that in hierarchical health systems, lower-level staff frequently see themselves solely as data producers, rather than as data users as well.^30 45^ When there are no constructive feedback loops in which data are used for learning and quality improvement, data lose their perceived relevance and are collected merely to fulfil top-down expectations, in what has been described as ‘empty compliance’.^46 47^

Our findings also give some indications as to how health workers’ sense of data ownership can shift when the modalities of data collection and feedback loops are altered. For the nurse-midwives directly involved with eRegistry data entry, their scope of practice changed as they had to triangulate data from different paper-based sources and piece together information along women’s continuum of care. Combined with the feedback on data trends provided by the ALERT team, this seemed to increase their sense of data ownership. Compared with the paper-based RHIS tools like the MTUHA registers, which have been primarily designed for health system management and planning purposes,^30^ the eRegistry seemed to enjoy greater perceived clinical relevance.

However, perceived relevance of data is merely a first step in promoting a data use culture for health system strengthening. Data feedback loops cannot exist in a vacuum: they are reliant on incentive structures, adequate infrastructure and human resources, as well as supportive supervision.^31 48^ In the case of the eRegistry, the potential for building such feedback loops was constrained by the limited involvement of leaders at the hospital, district and regional levels. Sociotechnical systems will never be ready off-the-shelf—they have to grow and evolve over time, with close involvement of the people whose work routines are being redesigned.^49^ As the eRegistry was not highly tailored to local data needs nor deeply embedded in local supervision and leadership structures, much of its potential to foster productive data use remained untapped. In line with recommendations by Wagenaar and colleagues,^48^ we consider close engagement with data producers and users crucial to develop future systems that better meet their needs. This includes making data available to them, building capacity for data use and supporting iterative quality improvement cycles.^48 50^ To promote long-term sustainability and avoid the added burden of parallel systems, we consider future digital health data systems in Tanzania to hold the greatest promise if they are owned and operated by the MoH or regional health offices. We also encourage researchers to reflect carefully in advance about the disruptions parallel data systems for research purposes may cause, and whether these disruptions can be justified by the potential benefits. Where possible, research should prioritise improving the functionality of existing RHIS to be adaptable for research and monitoring projects.

A methodological strength of our study was the combination of semi-structured interviews and focused ethnographic observation. Many studies on this topic rely only on interviews and focus group discussions, which may not fully capture non-articulated behaviours and underlying social dynamics surrounding collection and reporting of maternal and neonatal health data.^46^ The transferability of our findings is limited by the eRegistry’s project-specific nature. Nonetheless, we believe many of the key issues identified in this study are relevant to understand the complex nature of RHIS systems more generally, and that the insights are especially informative for future efforts to digitise such systems in Tanzania and other LMICs. Although JM, HTB and YK were not formally part of the ALERT study team, their linkage to the project may have caused a desirability bias in interviewees’ responses about the eRegistry. Additional reflections on positionality and power relations can be found in our reflexivity statement (online supplemental file 2).

## Conclusion

This study highlighted that digital solutions like the eRegistry have significant potential to enhance data quality and use in maternal and neonatal health. However, we must avoid a ‘techno-utopian’ view, treating these technologies as simple fixes. Experiences and perceptions of the eRegistry were closely tied to systemic issues like staffing, workload and infrastructure. The eRegistry increased a sense of data ownership among nurse-midwives involved in data entry, but the potential for feedback loops to improve care remained largely untapped. Our findings underline that digital data systems need to be deeply embedded and contextualised in order to provide accessible and relevant data that can be used for iterative improvements in quality of care.

## Supplementary material

10.1136/bmjgh-2024-016765 online supplemental file 1

10.1136/bmjgh-2024-016765 online supplemental file 2

## Data Availability

Making the full data set publicly available would breach the privacy that participants were promised. Therefore, we are unable to share full transcripts or field notes, but shorter excerpts (carefully reviewed for potential identifying details) may be shared with researchers who complete a data sharing agreement.
