# Supplementary material for: Data for whom? Experiences and perceptions of a perinatal eRegistry in two hospitals in Mtwara region, Tanzania
Source: BMJ Glob Health. 2024 Nov 20;9(11):e016765. doi: 10.1136/bmjgh-2024-016765 (PMC11580263; doi:10.1136/bmjgh-2024-016765)
Supplement: online supplemental file 1 [file bmjgh-9-11-s001.pdf]

Topic guides

These topic guides were used for the qualitative interviews conducted with different stakeholders in Mtwara region. Due to the iterative nature of qualitative research, the topic guides were adjusted over time to better fit the evolving nature of the research objectives and the insights gained in earlier stages of the research. Additional questions and edits were included directly in the Swahili versions while data collection was ongoing, but we made efforts to translate and include these in the English versions here. Please note that not all minor tweaks and variations are represented in the versions in this document.

The interviews were semi-structured, meaning the order of the questions was not fixed. The interviewers attempted to follow the flow of the participant's narrative as much as possible. Most of the probes included in the guides were typically not actually asked, as interviewees would have already covered this in their responses to previous, more general questions.

Language and phrasing were adapted to specific participants. Particularly for V3, questions were rephrased considerably to fit the professional position of the interviewee (i.e. whether they worked at hospital, district or regional level).

Table of Contents

Topic guides *1*

Topic guide V1 – For nurse-midwives directly involved with eRegistry data entry **2**

Topic guide V2 – For other labour ward staff **5**

Topic guide V3 – For managerial/administrative stakeholders **8**

Topic guide V1 – For nurse-midwives directly involved with eRegistry data entry

Briefing & introduction:
Thank you for agreeing to be interviewed. Before starting, I will give you an information sheet to read and a consent form to sign. Please take all the time you need to read through this and ask any questions you might have. I will ask you a series of open-ended questions to learn about your personal experiences and opinions. There are no right or wrong answers, and if there are any questions you prefer not to answer please let me know, and then we can move onto another question. The interview will take about 1 hour of your time. This is not an evaluation or a test: we value your opinions and your help is important.

Background
1. Can you please introduce yourself and tell me about your position here at the hospital?
2. Can you tell me about the ALERT activities you have been part of?

General characteristics of the eRegistry
Focus: get the participant to given an overview sources of the data that is entered in the eRegistry, and to describe the (daily) routine of collecting and entering it.
3. Can you tell me about how data for the perinatal eRegistry is collected at your hospital?
    - Where is the data first recorded before it is entered into the eRegistry?
4. Can you tell me about the daily routine of entering data into the eRegistry?
    - Can you describe the steps you take to enter the data?
    - At what moment in the day do you do it, and how long does it take?
5. Can you tell me about things or factors that help you to enter data in the perinatal eRegistry?
6. Can you tell me about things or factors that hinder you (make it challenging for you) to complete this task?
7. From your personal experience, how did the perinatal eRegistry change how information about mothers and babies is reported?
    - Which changes do you like?
    - Which changes do you dislike?
    - Before there was the eRegistry, was it harder or easier for you to document and report information?

Experiences and perceptions over time
Focus: getting the participant to reflect on changes in experiences and opinions about the perinatal eRegistry over time, from when it was first introduced until now.
8. I would like you to think back to when the perinatal eRegistry first started. Can you tell me about your initial personal experience when you first heard about the perinatal eRegistry?
    - What did you think when you first learned about the eRegistry during training?
    - What were the things that surprised you about the perinatal eRegistry?
    - What were specific things you struggled with or were confused about in the beginning?
    - How long did you need to get used to the registry?
    - How do you think the experience of your colleagues was when the perinatal eRegistry started? (Similar or different than your experience, why?)

9. What were the things that you wish had been included or explained more in the training, to make you personally feel more prepared?
   - Can you give some examples?
10. How did your experience with the eRegistry change over time?
    - In what ways is it different now than in the beginning?
    - Why do you think your experience is different now than before?

Quality of data

<u>Focus</u>: firstly explore the participant's own definition of 'good'/quality data, secondly explore different aspects of data quality (completeness, accuracy, timeliness) more specifically.

11. Let us think more generally about the data collected at the maternity ward, whether on paper or the data reported in the perinatal eRegistry. People often talk about the **quality** of data, but not everyone defines 'quality' in the same way. From your point of view, what makes data of good quality?
    - In an ideal case scenario, what are all the characteristics data should have to be of good quality?
    - When data is of good quality, how can it be used?
      - Who uses it?
      - Do you personally use it?
    - Do you think quality of data might be understood differently by other people?
      - For example: Do you think hospital leaders have the same view as you of what makes data of high quality?
      - Do you think officials at the district and regional level have the same view of what makes data of high quality?
12. Please, let us think about the **completeness** of data (the extent to which all patients are included, and all data variables are recorded). Can you think of reasons why data might be incomplete sometimes?
    - For example, what might be reasons that information about a particular woman is not recorded in the paper register?
    - For example, if there is an obstetric complication – what might be reasons that this is not recorded in the register?
13. Next, please let us think about the **accuracy** of data (if the information is correct and reflects exactly what happened). Can you think of issues that influence the accuracy of the data?
    - For example, what might be reasons that information recorded on a partograph does not reflect exactly what care a woman actually received?
14. Next, please let us think about the **timeliness** of data (whether it is available and shared when asked/required). Can you think of reasons why there might be delays in reporting of data?
    - Can you think of an example from your personal experience when there was an issue with the timeliness of data? What were the reasons for the delay?

Limits of the data

15. Thinking about the routine data collection on the ward (not just the eRegistry), is there any information you feel is important that is **not** captured in the data?

- Are there things which are important for women or their babies, which are not reflected in the data?
- If a person just looks at the data, what are things they might not understand about the services being provided?

Responsibilities, feedback & data use

Focus: in relation to the eRegistry, explore division of responsibilities, feedback/supervision and data use.

16. Can you explain how the responsibilities for entering data in the perinatal eRegistry are divided?
    - In your experience, is it always clear who is responsible?
    - In your view, is the work fairly divided?
17. How do your colleagues and superiors support you in using the perinatal eRegistry?
18. How do you get to hear about the findings from the eRegistry?
    - How is this (feedback) system working for you?
    - How do you think data from the perinatal eRegistry should be used? How can the data inform patient care and your work?
    - Is the data used to inform changes or decisions in your hospital?
19. Can you share any experiences of times when you used data from the eRegistry?
    - How did you go about making sense of the data? What information did the data give you, and how did you act upon it?
20. Are there any differences in the ways data is used now that the eRegistry is there, compared to before?
    - What are the reasons for these differences?

Long-term relevance and feasibility

Focus: Explore the participant's view on long-term relevance and feasibility of the eRegistry.

21. From your perspective, would you like to have an electronic register at your hospital in the long run?
    - Why (not)? What are the most important (dis)advantages?
22. To make it useful for you, what data would need to be collected in the long term?
23. Do you think it would be possible to keep the eRegistry running in the long term in this hospital?
    - Why (not)?
    - What are the main barriers to keeping the eRegistry running?
    - How could these barriers be addressed?

Wrapping up & debriefing

24. Do you have anything else you think would be relevant to mention or to add?

Thank you very much for taking the time for this interview. If you would like to stay informed about the project and our findings, I can note down your contact details and I will be in touch again.

Topic guide V2 – For other labour ward staff

Briefing & introduction:
Thank you for agreeing to be interviewed. Before starting, I will give you an information sheet to read and a consent form to sign. Please take all the time you need to read through this and ask any questions you might have. I will ask you a series of open-ended questions to learn about your personal experiences and opinions. There are no right or wrong answers, and if there are any questions you prefer not to answer please let me know, and then we can move onto another question. The interview will take about 1 hour of your time. This is not an evaluation or a test: we value your opinions and your help is important.

Background
1. Can you please introduce yourself and tell me about your position here at the hospital?
2. Can you tell me about the ALERT activities you have been part of?

General aspects of data collection and recording
<u>Focus</u>: to explore general experiences of data collection and reporting, as well as key barriers and facilitators.
3. I have noticed there are many different forms and registers where data is recorded about women and their babies. I am curious about your experiences with these and the different challenges they have. Can you tell me about the different forms and registers that are used on the ward? (can discuss each one separately)
    - Can you tell me about your daily routine of using these?
    - At what moment do you do this task? How much time does it take?
4. Can you tell me about things or factors that help you completing your documentation duties?
5. Can you tell me about things or factors that hinder you (make it challenging for you) to complete this task?
6. Can you explain how the responsibilities for documentation and reporting of data are divided?
    - In your experience, is it always clear who is responsible?
    - In your view, is the work fairly divided?
    - How do you and your colleagues work together to complete documentation and reporting tasks?

Quality (completeness, accuracy, timeliness)
<u>Focus</u>: firstly explore the participant's own definition of 'good'/quality data, secondly explore different aspects of data quality (completeness, accuracy, timeliness) more specifically.
7. People often talk about the **quality** of data, but not everyone defines 'quality' in the same way. From your point of view, what makes data collected about mothers and babies of good quality?
    - In an ideal case scenario, what are all the characteristics data should have to be of good quality?
    - Do you think quality of data might be understood differently by other people?
        - For example: Do you think hospital leaders have the same view as you of what makes data of high quality? Do you think officials at the district and regional level have the same view of what makes data of high quality?

8. Please, let us think about the **completeness** of data (the extent to which all patients are included, and all data variables are recorded). Can you think of reasons why data might be incomplete sometimes?
    - For example, what might be reasons that information about a particular woman is not recorded in the paper register? For example, if there is an obstetric complication – what might be reasons that this is not recorded in the register?
9. Next, please let us think about the **accuracy** of data (if the information is correct and reflects exactly what happened). Can you think of issues that influence the accuracy of the data?
    - For example, what might be reasons that information recorded on a partograph does not reflect exactly what care a woman actually received?
10. Next, please let us think about the **timeliness** of data (whether it is available and shared when asked/required). Can you think of reasons why there might be delays in reporting of data?
    - Can you think of an example from your personal experience when there was an issue with the timeliness of data? What were the reasons for the delay?

Limits of the data

11. Thinking about the routine data collection on the ward, is there any information you feel is important that is **not** captured in the data?
    - Are there things which are important for women or their babies, which are not reflected in the data?
    - If a person just looks at the data, what are things they might not understand about the services being provided?

Data use

12. Can you tell me about how the data you help collect and report are used?
    - Where do the data go, after they have been collected at the hospital?
    - Do you get feedback on the data?
    - What is this feedback like?
    - How is this (feedback) system working for you?
    - Are the data used to inform changes or decisions in your hospital?
13. Can you share any experiences of a time when you yourself, or your team, used data?
    - How did you go about making sense of the data? What information did the data give you, and how did you act upon it?
14. In an ideal scenario, how do you think data about women and babies should be used? How can the data inform patient care and your work?

Perinatal eRegistry

15. At the moment there is the perinatal eRegistry at your hospital. Can you tell me about your initial personal experience when you first heard about the perinatal eRegistry?
    - What did you think when you first learned about the eRegistry?
    - Were there things that surprised you about the perinatal eRegistry?
    - Were there specific things you struggled with or were confused about in the beginning?
16. From your personal experience, did the perinatal eRegistry change how information about mothers and babies is reported at your hospital?

- Which changes do you like? Which changes do you dislike?
- Are there any differences in the ways data is used now that the eRegistry is there, compared to before?

17. Can you think of reasons why there might be differences between the data in the paper registers and the eRegistry?
18. From your experience, how would you describe the quality of the data reported in the eRegistry?
    - What are the main strengths?
    - What are the main shortcomings?
    - What are the challenges which make it hard to record data of high quality? How could quality be improved?
19. From your perspective, would you like to have an electronic register at your hospital in the long run?
    - Why (not)? What are the most important (dis)advantages?
20. Do you think it would be possible to keep the eRegistry running in the long term in this hospital?
    - Why (not)?
    - What are the main barriers to keeping the eRegistry running?
    - How could these barriers be addressed?

Wrapping up & debriefing

Do you have anything else you think would be relevant to mention or to add?

Thank you very much for taking the time for this interview. If you would like to stay informed about my project and my findings, I can note down your contact details and I will be in touch again.

Topic guide V3 – For managerial/administrative stakeholders

Briefing & introduction:
Thank you for agreeing to be interviewed. Before starting, I will give you an information sheet to read and a consent form to sign. Please take all the time you need to read through this and ask any questions you might have. I will ask you a series of open-ended questions to learn about your personal experiences and opinions. There are no right or wrong answers, and if there are any questions you prefer not to answer please let me know, and then we can move onto another question. The interview will take about 1 hour of your time. This is not an evaluation or a test: we value your opinions and your help is important.

Background

1. Can you please introduce yourself and tell me about your position?
2. Can you tell me about the ALERT activities you have been part of?

General aspects of data collection and recording

<u>Focus</u>: to explore general experiences of routine data collection and reporting about women and babies, as well as key barriers and facilitators (<u>adjust phrasing</u> for higher-level stakeholders).

3. I have noticed there are many different forms and registers where data is recorded about women and their babies. I am curious about their purpose, their use and the different challenges they have. Can you tell me about the different forms and registers that are used at this hospital? (can discuss each one separately)
    - For whom (on whose behalf) are these data collected?
4. Can you explain how the responsibilities for documentation and reporting of data are divided the maternity ward at your hospital?
    - In your experience, is it always clear who is responsible?
    - In your view, is the work fairly divided?
5. Can you tell me about the challenges maternity care providers face in completing their documentation and reporting tasks? Can you tell me about things or factors that help care providers to complete their documentation and reporting tasks?
6. Can you tell me about the way you supervise and support care providers to complete their documentation duties?

Quality (completeness, accuracy, timeliness)

<u>Focus</u>: firstly explore the participant's own definition of 'good'/quality data, secondly explore different aspects of data quality (completeness, accuracy, timeliness) more specifically.

7. People often talk about the **quality** of data, but not everyone defines 'quality' in the same way. From your point of view, what makes data collected about mothers and babies of good quality?
    - In an ideal case scenario, what are all the characteristics data should have to be of good quality?
    - Do you think quality of data might be understood differently by other people? For example: Do you think health workers have the same view as you of what makes data of high quality?
    - Do you think officials at the district and regional level have the same view of what makes data of high quality?

8. Please, let us think about the **completeness** of data (the extent to which all patients are included, and all data variables are recorded). Can you think of reasons why data might be incomplete sometimes?
   - For example, what might be reasons that information about a particular woman is not recorded in the paper register? For example, if there is an obstetric complication – what might be reasons that this is not recorded in the register?
9. Next, please let us think about the **accuracy** of data (if the information is correct and reflects exactly what happened). Can you think of issues that influence the accuracy of the data?
   - For example, what might be reasons that information recorded on a partograph does not reflect exactly what care a woman actually received?
10. Next, please let us think about the **timeliness** of data (whether it is available and shared when asked/required). Can you think of reasons why there might be delays in reporting of data?

Can you think of an example from your personal experience when there was an issue with the timeliness of data? What were the reasons for the delay?

Limits of the data

11. Thinking about the routine data collection on the labour ward of your hospital (not just the eRegistry), is there any information you feel is important that is **not** captured in the data?
    - Are there things which are important for women or their babies, which are not reflected in the data?
    - If a person just looks at the data, what are things they might not understand about the services being provided?

Data use

12. Can you tell me about how the data collected about women and babies are used?
    - Where do the data go, after they have been collected at the hospital?
    - Do you get feedback on the data?
    - What is this feedback like?
    - How is this (feedback) system working for you?
13. Are the data used to inform changes or decisions in your hospital?
    - Can you share any experiences of a time when you yourself, or your team, used data?
    - How did you go about making sense of the data? What information did the data give you, and how did you act upon it?
    - In an ideal scenario, how do you think data about women and babies should be used? How can the data inform patient care and your work?

Perinatal eRegistry

14. At the moment there is the perinatal eRegistry at your hospital. Can you tell me about your initial personal experience when you first heard about the perinatal eRegistry?
    - What did you think when you first learned about the eRegistry?
    - Were there things that surprised you about the perinatal eRegistry?
    - Were there specific things you struggled with or were confused about in the beginning?
15. From your personal experience, did the perinatal eRegistry change how information about mothers and babies is reported at your hospital?

- Which changes do you like? Which changes do you dislike?
- What do you see as the benefits of the eRegistry for your work?

16. Are there any differences in the ways data is used now that the eRegistry is there, compared to before?
17. Can you think of reasons why there might be differences between the data in the paper registers and the eRegistry?
18. From your experience, how would you describe the quality of the data reported in the eRegistry?
    - What are the main strengths?
    - What are the main shortcomings?
    - What are the challenges which make it hard to record data of high quality? How could quality be improved?
19. From your perspective, would you like to have an electronic register at your hospital in the long run?
    - Why (not)? What are the most important (dis)advantages?
20. Do you think it would be possible to keep the eRegistry running in the long term in this hospital?
    - Why (not)?
    - What are the main barriers to keeping the eRegistry running?
    - How could these barriers be addressed?

Wrapping up & debriefing

Do you have anything else you think would be relevant to mention or to add?

Thank you very much for taking the time for this interview. If you would like to stay informed about my project and my findings, I can note down your contact details and I will be in touch again.
