# Supplementary material for: Data for whom? Experiences and perceptions of a perinatal eRegistry in two hospitals in Mtwara region, Tanzania
Source: BMJ Glob Health. 2024 Nov 20;9(11):e016765. doi: 10.1136/bmjgh-2024-016765 (PMC11580263; doi:10.1136/bmjgh-2024-016765)
Supplement: online supplemental file 2 [file bmjgh-9-11-s002.pdf]

**Reflexivity statement**

This statement addresses the questions and domains suggested in the *Consensus statement on measures to promote equitable authorship in the publication of research from international partnerships*. We have also added additional reflections on positionality and power relations that we considered of relevance.

1. **Study conceptualisation**

JM, the first author of the study, is a PhD student with Dutch nationality who is based in Belgium and LB, JVO, CH and AP are her PhD supervisors. JM developed the overall study protocol with input from her supervisors. All co-authors were involved at different stages of the study's design, data collection, data analysis and write-up. JM developed the topic guides for the study, with input from LB, JVO, MA, CH and AP.

As outlined in our paper's introduction, the Tanzanian government implemented a Data Dissemination and Use Strategy for its health sector in 2015, as well as a National Data Quality Guideline in 2016 (Darcy et al., 2017; HSSP, 2021). In its 2021 Health Sector Strategic Plan (HSSP), the Ministry of Health expressed the ambition that "the HMIS should become the basis for evidence-based planning and management from the lowest level to the top level in the health sector" (HSSP, 2021, p. 71). The study focus thus aligns with current priorities pursued by the government of Tanzania. Although the e-registry was implemented in the context of the quality improvement and implementation science project ALERT (https://alert.ki.se/), the qualitative insights have broader relevance for ongoing efforts to digitise Tanzania's RHIS.

2. **Research management**

Data collection for this study was funded through JM's PhD fellowship from the Flanders research foundation (FWO), reference number 1130323N. The study was linked to the ALERT project, which has supported several PhD positions in partner institutions in LMICs.

3. **Data acquisition and analysis**

Every individual involved in data collection is either credited as a co-author or acknowledged by name along with their contributions. During data collection, daily debrief meetings between data collectors, as well as online discussions with other team members, informed preliminary analyses and further data collection (e.g. through topic guide adjustments and additions). When data collection was concluded, debrief discussions among members of the research team facilitated collaborative interpretation.

4. **Data interpretation**

With regards to data analysis, initial codes were identified by JM through refamiliarization with the whole data set. Following double-coding of a subset of interview transcripts by JM and AK, a shared coding tree was developed. This codebook was used to re-code all transcripts and field notes in NVivo by JM, with codes being refined and renamed along the course of coding. A selection of relevant quotes and extracts per code was translated into English to facilitate discussion within the author team. Feedback and input from the team informed the structure of the eventual write-up. Co-authors who are native Swahili speakers cross-checked the translations from Swahili into English.

5. **Drafting and revising for intellectual content**

Manuscript writing was led by JM, with input from team members at different points in the process. Prior to writing the full manuscript, JM developed a paper outline which co-authors provided input on. After JM had prepared a full draft, all co-authors gave written feedback. JM

also had discussions with her PhD supervisors about the progress of the manuscript. In some cases, JM sought input and comments from individuals via direct messaging to encourage junior team members to review and revise the manuscript.

This study will be published open access. The findings will also be disseminated at scientific conferences and during stakeholder meetings in Tanzania at hospital, district, regional and national levels.

### 6. Authorship

Our research team included members from both high income and low-middle income countries. The primary affiliations of JM, LB, JVO, CH are with institutions in Europe, while the primary affiliations of AK, YK, HTB, MA and AP are with institutions in Tanzania. The research team consisted of five women and four men and therefore had a good gender balance. Contributions per author are listed below:

- JM: conceived and designed the methodology of the study; collected data; performed analysis and was responsible for drafting and revising the manuscript.
- AK: coded a subset of interviews; contributed to development of the coding tree; provided feedback on the manuscript.
- YK: led the interviews at hospital B (with JM present); took part in debrief discussions; provided feedback on the manuscript.
- HTB: led the interviews at hospital A (with JM present); took part in debrief discussions; provided input on the manuscript.
- LB: supervised conceptualisation and methodology of the study; took part in debrief discussions; provided feedback on the manuscript.
- JVO: supervised conceptualisation and methodology of the study; took part in debrief discussions; provided feedback on the manuscript.
- CH: supervised conceptualisation and methodology of the study; provided feedback on the manuscript.
- MA: supervised conceptualisation and methodology of the study; supported research activity planning and execution; took part in debrief discussions; provided feedback on the manuscript.
- AP: supervised conceptualisation and methodology of the study; supported research activity planning and execution; took part in debrief discussions; provided feedback on the manuscript.

### 7. Training

This smaller study did not include formal training of LMIC researchers. However, de-briefing sessions with senior researchers present provided learning opportunities for junior researchers. In addition, within the larger ALERT project there were various opportunities for attending (research methodology) trainings and conferences for LMIC researchers.

### 8. Infrastructure

The study has not directly contributed to improvements in infrastructure.

### 9. Governance & safeguarding

Various safeguarding procedures were used to protect study participants. We have chosen not to name the districts and hospitals in which this research was conducted, to minimize the possibility that findings and quotes can be linked to specific locations or individuals. Written informed consent was obtained from all interview participants. Participants were reimbursed for their time in line with per diem rates put forward by the Tanzanian government, which amounted to approximately 7 EUR/USD per interview. Participants had the choice to not answer

(particular) interview questions, take a pause, or discontinue the study at all times, and were informed of this. All participants' names were replaced by numerical IDs to protect confidentiality, and the link between names and IDs was stored in a password-protected encrypted file.

All researchers had sufficient time and opportunity to provide input and review the manuscript, and potential power asymmetries among junior and senior researchers were navigated through one-to-one communication in some cases, including through more informal channels (e.g. WhatsApp).

10. **Additional reflections on positionality and power relations**

The researchers' background and positions affected the angle of investigation that was chosen; the methods that were selected; the findings that were considered relevant to publish; and how they were framed in the final manuscript. Positionality and power relations also affected how the researchers were viewed by study participants. This was particularly relevant during the observations carried out by JM, the European first author. JM had previously spent time in Tanzania during her MSc studies and as a research assistant, but this study was her first data collection stay in Tanzania for her doctoral research. JM speaks intermediate-level Swahili. As a non-Tanzanian woman without medical training, it is fair to say she was considered an outsider by the study participants. When asked, she would help out with small tasks such as fetching supplies or carrying patients' belongings when they were transferred to/from another ward. Not being a medical professional made it easier to justify why she stayed strictly out of clinical tasks. Being of a similar age to many of the nurse-midwives seemed to help build rapport and reduce power differentials.

JM's positionality played out differently in the two hospitals. At hospital A, labour ward staff were used to having interns and students coming for rotations and clinical observations, including non-Tanzanian students. Her presence was therefore not completely out of the ordinary, although her interest in routine health data was somewhat unusual. At hospital B, on the other hand, foreign visitors were rare and the concept of observation was less familiar to the health facility staff. Consequently, it took more time to explain the purpose of the observations and staff were more surprised by JM's refusal to take part in clinical tasks. Being accompanied by HTB or YK for the first introductions to hospital staff helped ensure that the objectives of the observations and interviews were clearly communicated. This included making it clear to participants that the researchers by no means aimed to identify 'incorrect' reporting practices, nor blame specific individuals or institutions. Nonetheless, it is possible that JM's presence on the ward impacted the way people went about their daily routines, including the way they engaged with routine health data and the e-registry.

With regards to the interviews, it became clear over the course of the research that a recorded interview format impacted the way people some people responded to questions. There were labour ward staff who were talkative and spontaneous in everyday interactions, yet were more reserved during their interview. Conversely, there were also interview participants who visibly enjoyed the opportunity to speak more at length, and who touched upon many issues in their interview that had not come up in informal conversations. Generally, we felt the observations and interviews enriched each other significantly. Interview participants were able to refer to events and routines on the ward which JM had observed ("As you've seen…"), and the observations allowed for an in-depth contextual understanding of the topics discussed in the interviews.
